# Clinical Implications and Molecular Characterization of Drebrin-Positive, Tumor-Infiltrating Exhausted T Cells in Lung Cancer

**DOI:** 10.3390/ijms232213723

**Published:** 2022-11-08

**Authors:** Kosuke Imamura, Yusuke Tomita, Ryo Sato, Tokunori Ikeda, Shinji Iyama, Takayuki Jodai, Misako Takahashi, Akira Takaki, Kimitaka Akaike, Shohei Hamada, Shinya Sakata, Koichi Saruwatari, Sho Saeki, Koei Ikeda, Makoto Suzuki, Takuro Sakagami

**Affiliations:** 1 Department of Respiratory Medicine, Faculty of Life Sciences, Kumamoto University, Honjo 1-1-1, Chuo-ku, Kumamoto-shi 860-8556, Kumamoto, Japan; 2 Department of Respiratory Medicine, Kumamoto University Hospital, Honjo 1-1-1, Chuo-ku, Kumamoto-shi 860-8556, Kumamoto, Japan; 3 Laboratory of Stem Cell and Neuro-Vascular Biology, Cell and Developmental Biology Center, National Heart, Lung, and Blood Institute, National Institutes of Health, 10 Center Drive, Bethesda, MD 20892, USA; 4 Laboratory of Clinical Pharmacology and Therapeutics, Faculty of Pharmaceutical Sciences, Sojo University, 4-22-1, Ikeda Nishi-ku, Kumamoto-shi 860-0082, Kumamoto, Japan; 5 Department of Thoracic and Breast Surgery, Faculty of Life Sciences, Kumamoto University, Honjo 1-1-1, Chuo-ku, Kumamoto-shi 860-8556, Kumamoto, Japan

**Keywords:** drebrins, lymphocytes, tumor-infiltrating, lung neoplasms, tumor microenvironment, prognosis

## Abstract

T cells express an actin-binding protein, drebrin, which is recruited to the contact site between the T cells and antigen-presenting cells during the formation of immunological synapses. However, little is known about the clinical implications of drebrin-expressing, tumor-infiltrating lymphocytes (TILs). To address this issue, we evaluated 34 surgical specimens of pathological stage I–IIIA squamous cell lung cancer. The immune context of primary tumors was investigated using fluorescent multiplex immunohistochemistry. The high-speed scanning of whole-slide images was performed, and the tissue localization of TILs in the tumor cell nest and surrounding stroma was automatically profiled and quantified. Drebrin-expressing T cells were characterized using drebrin^+^ T cells induced in vitro and publicly available single-cell RNA sequence (scRNA-seq) database. Survival analysis using the propensity scores revealed that a high infiltration of drebrin^+^ TILs within the tumor cell nest was independently associated with short relapse-free survival and overall survival. Drebrin^+^ T cells induced in vitro co-expressed multiple exhaustion-associated molecules. The scRNA-seq analyses confirmed that the exhausted tumor-infiltrating CD8^+^ T cells specifically expressed drebrin. Our study suggests that drebrin-expressing T cells present an exhausted phenotype and that tumor-infiltrating drebrin^+^ T cells affect clinical outcomes in patients with resectable squamous cell lung cancer.

## 1. Introduction

Lung cancer is the most frequent cause of cancer-related death worldwide [1]. Early-stage non-small-cell lung cancer (NSCLC) is generally treated with complete surgical excision. Approximately 30–40% of patients with NSCLC present with resectable disease [2]. However, 30–55% of patients show disease recurrence within five years after surgery [3]. The five-year survival rate of surgically resected patients ranges from 92% in stage IA to 36% in stage IIIA according to the eighth tumor, node, and metastasis (TNM) system [4]. Patients with lung cancer who undergo surgical treatment have a variable prognosis; therefore, current clinicopathological staging cannot fully predict postoperative recurrence, which results in an urgent need to establish biomarkers so as to identify patients at a comparatively higher risk of postoperative recurrence and cancer-related death.

The tumor immune microenvironment (TIME) evolves during tumor growth and dissemination [5,6,7]. TIME differs not only between cancer types [8] but also between patients with the same disease [9]. TIME compositions, such as the types of immune cells, density, and locations within the tumors influence the tumor characteristics and clinical outcomes [5,10,11]. Understanding TIME is essential for developing prognostic and predictive biomarkers, identifying novel therapeutic targets, and implementing long-term management strategies for NSCLC patients with resectable disease [12].

A notable constituent of TIME is the tumor-infiltrating lymphocytes (TILs) [13]. TILs have been studied as prognostic and/or predictive immune biomarkers for NSCLC [14]. The immune context is defined by the density and location of the TILs [6,7]. The quantification of the spatial patterns of TILs in tumor regions have a significant prognostic value [15,16,17].

To identify and quantify TILs, multiplexed immunofluorescence (mIF)- or immunohistochemistry (mIHC)-based analyses of tissue sections have been employed [18]. However, most mIF/IHC methods are based on visual estimation, lacking objectivity [13]. Therefore, the development of an automated method for the objective quantification of TILs is necessary. In addition, current imaging techniques and computer technologies can digitize histology slides, thereby enabling the automated analysis of whole-slide images of tissue slides, which offers unique opportunities to quantify the spatial architectural patterns and discover novel prognostic and predictive markers [19].

Drebrin was first described as an actin-binding protein [20]. Drebrin is expressed in immune cells such as the T cells [21], mast cells [22], and dendritic cells [23]. Drebrin is recruited to the contact site between the T cells and antigen-presenting cells during the formation of immunological synapses and modulates T cell activation [21]. Therefore, we hypothesized that drebrin-expressing TILs may play a predictive or prognostic role in postoperative lung cancer patients.

In this study, we investigated the clinical implications of drebrin^+^ TILs in patients with lung cancer by immunohistochemically stained tissue sections from 34 surgically treated patients with squamous cell lung cancer (Sq). Moreover, we characterized drebrin^+^ T cells using drebrin^+^ T cells induced in vitro. Furthermore, we conducted an in silico analysis and examined the expression of the drebrin gene (*drebrin1*) in NSCLC patients. Our study suggests that drebrin^+^ T cells have exhausted T cell signatures and impact the clinical outcomes of patients with resectable lung cancer.

## 2. Results

### 2.1. Drebrin Expression in Peripheral and Tumor-Infiltrating T Lymphocytes

**Figure 1:**
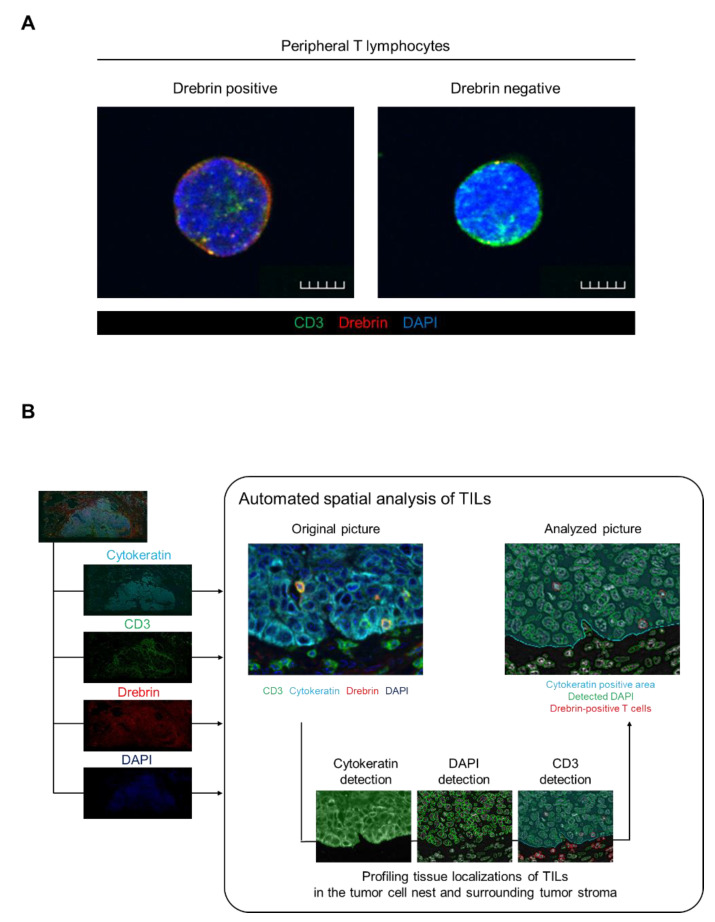
Drebrin expression detected in peripheral T cells and schematics of TIL analysis among patients with primary squamous cell cancer. (**A**) Representative images of the peripheral T cells. Purified T cells from healthy donors’ peripheral blood were stained with CD3 (green), drebrin (red), and DAPI (blue). Scale bars, 50 µm. (**B**) Schematics of the automated spatial analysis of the TILs. Surgical tissue specimens from patients with squamous cell lung cancer (N = 34) were stained by multiplex fluorescent immunohistochemistry, and images of all the tumor areas were analyzed. TIL, tumor-infiltrating lymphocyte.

T lymphoblasts and Jurkat J77 cells express drebrin. However, little is known about drebrin expression in human peripheral T lymphocytes and the clinical significance of drebrin-expressing T cells in cancer patients. We evaluated the drebrin expression of CD3^+^ T cells in human peripheral blood using immunocytochemistry analysis. Consistent with previous reports [21,24], drebrin was found to be distributed throughout the subcortical regions of the T cell membrane and cytoplasm (Figure 1A). We investigated the frequency of drebrin-expressing T cells in the peripheral blood samples of healthy donors. Most of peripheral T cells did not express drebrin. Few drebrin^+^ T cells were detected in the circulating T cells. The median value of the drebrin^+^ cells among the circulating CD3^+^ T cells was 1.78%.

**Figure 2:**
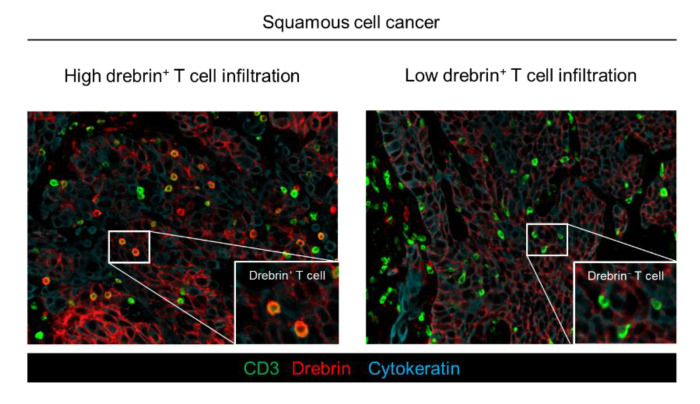
Distinct patterns of drebrin-expressing TILs among lung cancer patients. Representative images of drebrin^+^ TILs from two patients diagnosed at the same pathological stage. Pan-cytokeratin (blue) of tumor cells, CD3 (green), and drebrin (red) were simultaneously stained. Drebrin^+^ or drebrin^−^ TILs are shown at a high magnification. TILs, tumor-infiltrating lymphocytes.

Drebrin is recruited to the contact site between the T cells and antigen-presenting cells during the formation of immunological synapses and modulates T cell activation. Therefore, we hypothesized that TILs may express drebrin through tumor antigen stimulation, and that drebrin-expressing TILs may affect clinical outcomes in lung cancer patients. Fluorescent mIHC analysis has been shown to capture multidimensional data related to the tissue architecture, spatial distribution of tumor-infiltrating immune cells, and co-expression of signaling [25]. We investigated surgically resected primary tumors of patients with Sq using mIHC. The pan-cytokeratin of the tumor cells, CD3, drebrin, and nuclei were simultaneously stained. The high-speed scanning of whole-slide images was performed on stained tissue sections, and the tissue localization of the TILs was evaluated. CD3^+^ T cells, CD3^+^/drebrin^+^ T cells, and CD3^+^/drebrin^−^ T cells in the tumor cell nest and surrounding stroma were profiled and quantified by an automated quantitative pathology imaging system [6,7]. The tissue localization of TILs in the tumor cell nest and surrounding tumor stroma was profiled according to their relationship with cytokeratin-positive tumor cells (Figure 1B). Drebrin-expressing TILs were detected in the primary Sq tumors. The density of the tumor-infiltrating drebrin^+^ T cells varied according to the tumor. We found that primary lung tumors had distinct patterns of drebrin^+^ TILs, despite the patients having the same pathological stage (Figure 2). Therefore, we hypothesized that drebrin-expressing TILs may affect clinical outcomes and further investigated the role of drebrin^+^ T cells in primary lung tumors.

### 2.2. Association of Drebrin-Expressing TILs in Tumor Cell Nest with Survival Outcomes in Patients with Lung Cancer

We evaluated the clinical implications of drebrin-expressing TILs in patients with resectable Sq. The baseline characteristics of the 34 patients who underwent curative surgery are shown in Table 1. The patients were stratified by the median number of drebrin^+^ T cells in the tumor cell nests per tumor area (high or low drebrin^+^ T cell infiltration).

**Figure 3:**
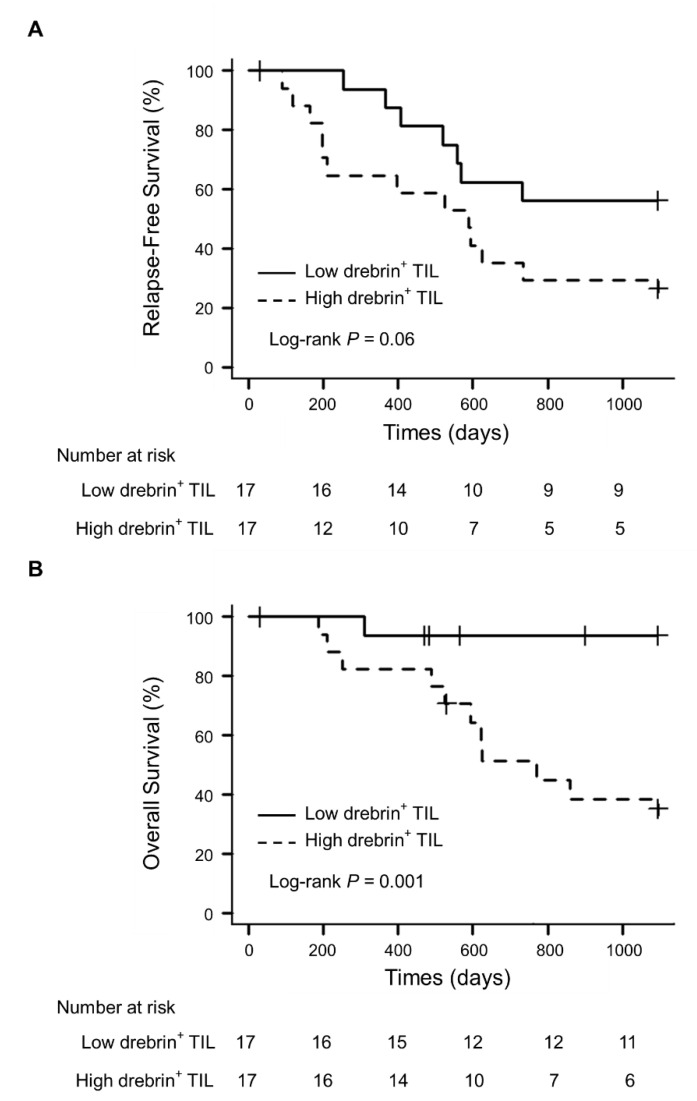
Association of drebrin-expressing TILs in tumor cell nests and survival outcomes. (**A**) Relapse-free survival of patients with high and low infiltrations of drebrin^+^ TILs. (**B**) Overall survival of patients with high and low infiltrations of drebrin^+^ TILs. TILs, tumor-infiltrating lymphocytes.

No statistical differences in age, sex, smoking history, pathological stage, mutation status of the epidermal growth factor receptor (EGFR), and microinvasion were evident between the patients with high or low drebrin^+^ T cell infiltration into the tumor cell nests. Survival analyses were conducted using univariate analyses and multivariate Cox proportional hazard regression models, using propensity scores to correct potential confounding factors that may affect the treatment assignment. The overall number of CD3^+^ T cells did not affect the survival (data not shown). However, the univariate survival analyses confirmed that the patients with high drebrin^+^ T cell infiltration showed a trend toward a short RFS (hazard ratio (HR), 2.40; *p* = 0.06; 95% confidence interval (CI), 0.95–6.02) (Figure 3A). Moreover, high drebrin^+^ T cell infiltration was significantly associated with a short OS of the patients (HR, 13.46; *p* = 0.001; 95% CI, 1.73–104.70) (Figure 3B).

Next, we applied multivariate Cox proportional hazard models with IPTW, and propensity scores were estimated using a logistic regression model for multivariate modeling. Age, sex, smoking history, pathological stage (I–II or III), adjuvant therapy, pleural invasion, lymphatic invasion, vascular invasion, and drebrin^+^ tumor cells were used as the background factors. The propensity score analysis confirmed that high drebrin^+^ T cell infiltration in the patients was significantly associated with a short RFS (IPTW-adjusted HR, 3.06; *p* = 0.012; 95% CI, 1.28–7.31) and OS (IPTW-adjusted HR, 29.17; *p* = 0.002; 95% CI, 3.55–239.91). We further confirmed the statistical robustness, ensuring that high drebrin^+^ T cell infiltration in the patients was independently associated with a short RFS (HR, 4.07; *p* = 0.034; 95% CI, 1.11–14.92) and OS (HR, 47.75; *p* = 0.001; 95% CI, 4.80–475.31).

### 2.3. Long-Term T Cell Stimulation Increases Drebrin Expression

**Figure 4:**
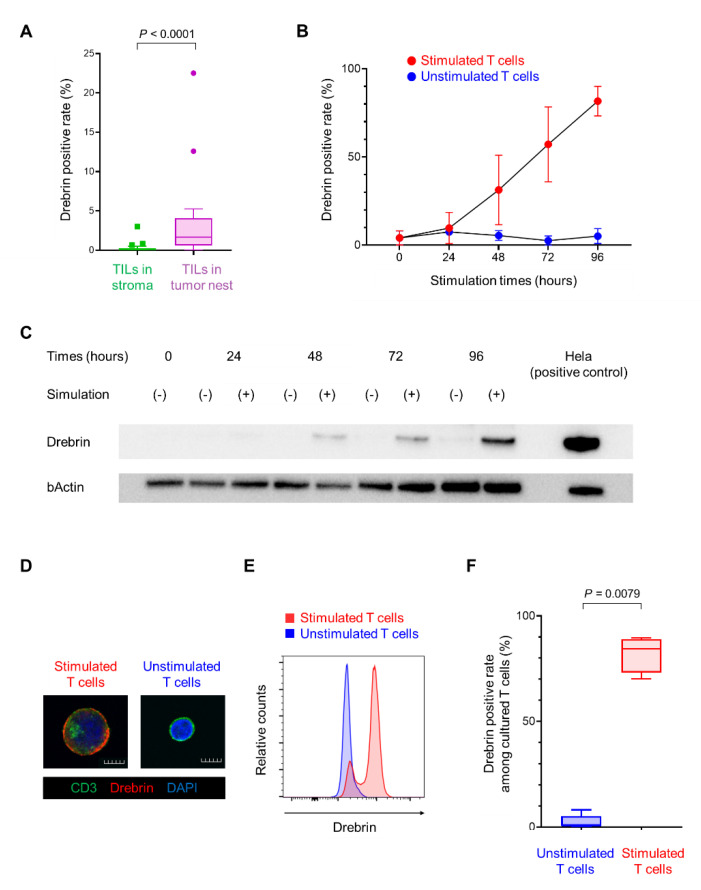
Long−term T cell stimulation increases drebrin expression. (**A**) Frequencies of drebrin^+^ TILs in tumor cell nests or the surrounding stroma. (**B**) Assessment of drebrin expression according to the culture period with or without stimulation by flow cytometric analysis. (**C**) Drebrin expression of each culture period by western blot analysis. Hela cells were used as a positive control. (**D**) Representative images of stimulated or unstimulated T cells (96 h) stained with CD3 (green), drebrin (red), and DAPI (blue). Scale bars, 50 µm. (**E**) Representative histograms of stimulated or unstimulated T cells (96 h) by flow cytometric analysis. (**F**) Frequency of drebrin^+^ T cells among stimulated or unstimulated T cells (96 h). TILs, tumor-infiltrating lymphocytes.

To elucidate the link between high drebrin^+^ T cell infiltration and poor clinical outcomes, we next characterized the drebrin^+^ T cells. NSCLC patients with abundant exhausted TILs have a short disease-free survival after surgical resection [26]. Therefore, we hypothesized that drebrin expression in the T cells may be involved in T cell exhaustion. Notably, the drebrin-expressing TILs in the tumor cell nest were significantly higher than those in the surrounding stroma (Figure 4A), suggesting that the stimulation of tumor antigens through direct contact between the tumor and T cells might have influenced the drebrin expression in TIME. First, we investigated the changes in drebrin expression in the peripheral T cells after their long-term stimulation in vitro. The purified CD3^+^ T cells from healthy donors were incubated with CD3/CD28/CD2 beads to stimulate the T cells and were cultured for a long period of time. The drebrin expression in the purified CD3^+^ T cells increased upon in vitro stimulation according to the culture period (Figure 4B,C). The number of drebrin-expressing CD3^+^ T cells was significantly higher under in vitro stimulation than unstimulated conditions (Figure 4D–F), indicating that T cell activation can induce drebrin expression in CD3^+^ T cells.

### 2.4. Drebrin^+^ T Cells Co-Express Multiple Exhaustion-Associated Molecules

**Figure 5:**
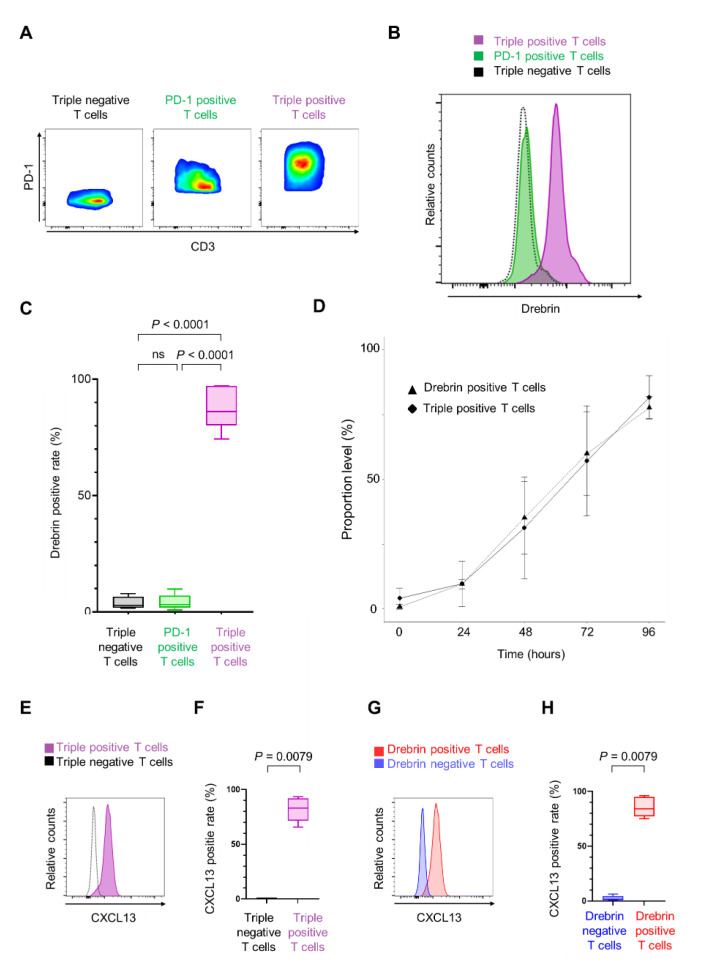
Drebrin^+^ T cells co-express multiple exhaustion-associated molecules. (**A**) Representative flow cytometry plots of three populations. Triple-negative T cells are negative for PD-1, TIM-3, and LAG-3. PD-1 positive T cells are positive for PD-1 but negative for TIM-3 and LAG-3. Triple-positive T cells are positive for PD-1, TIM-3, and LAG-3. Cells were cultured for 96 h. (**B**) Representative histograms of each population. (**C**) Frequencies of drebrin^+^ T cells among each population. (**D**) A correlation between drebrin expression in T cells and triple-positive T cells under in vitro culture conditions. Cells were cultured for up to 96 h. (**E**) Representative histograms of triple-positive or triple-negative T cells. (**F**) Frequencies of CXCL13^+^ T cells among each population. Cells were cultured for 96 h. (**G**) Representative histograms of drebrin-positive or drebrin-negative T cells. (**H**) Frequencies of CXCL13^+^ T cells among each population. Cells were cultured for 96 h. CXCL13, chemokine ligand 13; LAG-3, lymphocyte activation gene 3; PD-1, programmed cell death-1; TIM-3, T cell immunoglobulin- and mucin-domain-containing protein 3.

Exhausted TILs co-express activation-associated molecules, revealing an indispensable relationship between T cell activation and exhaustion [26,27]. Therefore, we investigated the relationship between drebrin and exhaustion-associated molecules, including programmed cell death-1 (PD-1), T cell immunoglobulin- and mucin-domain-containing protein 3 (TIM-3), and lymphocyte activation gene 3 (LAG-3), in CD3^+^ T cells during in vitro stimulation. The drebrin expression in triple-positive T cells (positive for PD-1, TIM-3, and LAG-3) was higher than that in PD-1 single-positive T cells (positive for PD-1, and negative for TIM-3 and LAG-3) and triple-negative T cells (negative for PD-1, TIM-3, and LAG-3) (Figure 5A–C).

Two analytical steps were performed to investigate the relationship between drebrin expression in T cells and triple-positive T cells. A significant correlation was found between the portion of drebrin^+^ T cells and that of triple-positive T cells (r = 1.00; *p* = 0.017) (Figure 5D), indicating that drebrin expression in T cells is associated with the T cells’ co-expression of PD-1, TIM-3, and LAG-3.

Chemokine ligand 13 (CXCL13) is significantly upregulated in highly exhausted TILs [28,29,30,31]. Therefore, we investigated the CXCL13 expression in the T cells. The number of CXCL13^+^ T cells was significantly higher in the triple-positive T cells than in the triple-negative T cells (Figure 5E,F). Additionally, the drebrin^+^ T cells expressed significantly higher levels of CXCL13 than the drebrin^−^ T cells (Figure 5G,H).

### 2.5. Single-Cell Transcriptional Characterization of Drebrin^+^ T Cells in NSCLC Patients

**Figure 6:**
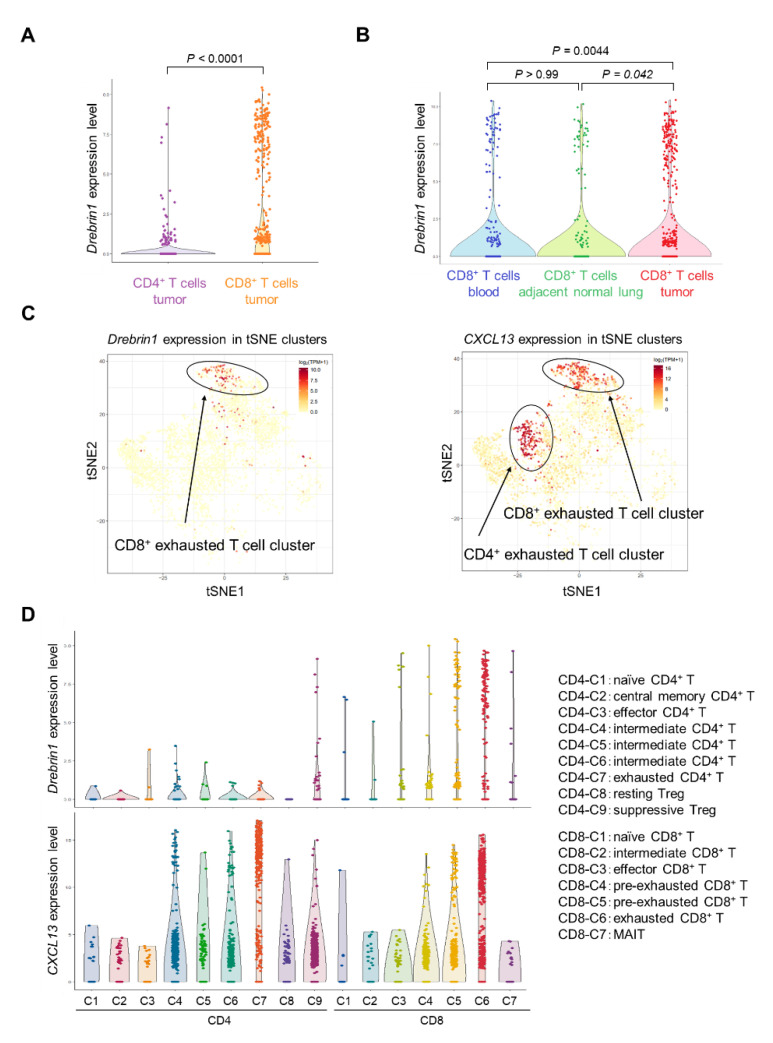
Transcriptional characterization of drebrin^+^ T cells in NSCLC patients. (**A**) Comparison of *drebrin1* levels between CD4^+^ and CD8^+^ T cells. Each dot represents one cell. (**B**) Comparison of *drebrin1* levels between different tissue samples. Each dot represents one cell. (**C**) Expressions of *drebrin1* and *CXCL13* in T cells from the tumor are illustrated in the t-SNE plots. Each dot represents one cell. (**D**) Expressions of *drebrin1* and *CXCL13* in each cluster are illustrated in violin plots. Each dot represents one cell. The definition of each cluster is indicated on the right panel. Intermediate cells represent cells bridging naïve, effector, and exhausted clusters. MAIT, mucosal-associated invariant T cells; NSCLC, non-small-cell lung cancer; t-SNE, t-distributed stochastic neighbor embedding.

We analyzed single-cell data from a published database on the T cells of NSCLC patients [32]. The *drebrin1* expression in isolated T cells from the peripheral blood, adjacent normal tissue, and tumors was investigated at the single-cell level. The *drebrin1* expression in T cells from the peripheral blood was mainly observed in the CD8^+^ T cells (Supplementary Figure S1). In addition, *drebrin1* was more abundant in the tumor-infiltrating CD8^+^ T cells than in the CD8^+^ T cells in the peripheral blood and adjacent normal tissues (Figure 6A,B). *Drebrin1*-expressing cells were most prominently represented in the exhausted tumor-infiltrating CD8^+^ T cell clusters at the single-cell level (Figure 6C,D), although CXCL13 was expressed in the exhausted tumor-infiltrating CD4^+^ and CD8^+^ T cell clusters (Figure 6C,D). PD-1 (*PDCD1*), TIM-3 (*HAVCR2*), and LAG-3 (*LAG3*) were broadly expressed in all the T cell clusters (Supplementary Figure S2). We analyzed another published database on NSCLC patients [31], confirming that *drebrin1* expression was observed in TILs, with a strong expression of exhaustion-associated molecules, including PD-1, TIM-3, LAG-3, and CXCL13.

Taken together, drebrin^+^ T cells co-expressed multiple exhaustion-associated molecules, including PD-1, TIM-3, LAG-3, and CXCL13, and drebrin was selectively expressed in exhausted tumor-infiltrating CD8^+^ T cells, suggesting that drebrin is a novel exhaustion-associated molecule.

## 3. Discussion

This study is the first to report the prognostic value of drebrin-expressing TILs in patients with resectable Sq. We showed that drebrin^+^ T cells induced in vitro co-expressed multiple exhaustion-associated molecules; however, drebrin was barely expressed in PD-1 single-positive T cells. Moreover, the transcriptional profile of the drebrin^+^ TILs in patients with NSCLC confirmed that the drebrin^+^ T cells were associated with exhausted CD8^+^ T cells in the tumors.

The computer-based automated quantification of TILs has helped to mitigate subjectivity and improve the low reproducibility of human-based quantification [16]. Moreover, the spatial location of immune cells is useful for predicting the patient prognosis [33,34]. In our study, automated quantification enabled the objective analysis of whole-slide images, and we profiled and quantified the tissue localization of TILs in the tumor cell nests and the surrounding stroma. This automated spatial analysis of the TILs in whole-slide images revealed the prognostic value of the tumor-infiltrating drebrin^+^ T cells in postoperative patients with Sq.

Patients with progressive T cell exhaustion in the TILs have a short disease-free survival after the surgical resection of NSCLC [26]. We found that a high density of drebrin-expressing TILs was independently associated with a short RFS and OS. In addition, drebrin^+^ T cells had the exhausted T cell signatures. Our findings suggest that the drebrin-expressing TILs observed in patients with lung cancer represent exhausted T cells; therefore, patients with a high infiltration of drebrin^+^ TILs had a short RFS and OS.

The formation of the immune synapses and subsequent T cell activation are highly dependent on actin cytokeratin, and drebrin plays a relevant functional role in the T cells during the generation of immune responses [21]. In TIME, T cells are exposed to chronic antigen stimulation and enter a state of dysfunction, which is known as T cell exhaustion. In the current study, more drebrin^+^ T cells were detected in the tumor cell nests than in the surrounding stroma, suggesting a possible relationship between chronic antigen stimulation by cancer cells and the induction of drebrin expression in the T cells of tumor cell nests.

Immune checkpoint inhibitors (ICIs) have substantially improved survival outcomes [35,36,37]. Currently, adjuvant ICI therapy has been investigated in several clinical trials to assess whether ICIs can improve outcomes after curative surgery as an adjuvant therapy. The targeting of immune checkpoint receptors has been performed to reinvigorate exhausted T cells [38,39]. Therefore, the status of T cell exhaustion has the potential to act and be evaluated as a prognostic and/or predictive biomarker in adjuvant settings for lung cancer patients [26]. CXCL13 is an important factor involved in the recruitment of immune cells to the tumor microenvironment and serves as a key molecular determinant during the formation of tertiary lymphoid structures [40]. It has been revealed that TILs secret CXCL13 [41]. Several studies investigating TILs in human malignancies have identified significant CXCL13 upregulation in highly exhausted TILs [28,29,30]. Moreover, CXCL13 was identified as the best marker of the intrinsic features of T cells in a study involving NSCLC patients [42]. CXCL13^+^ T cells are associated with poor clinical outcomes and an excellent response to ICI therapy. Drebrin^+^ T cells induced in vitro co-expressed high levels of CXCL13, and our in silico analysis revealed an association between the expression of *drebrin1* and *CXCL13* in the TILs, suggesting that drebrin^+^ TILs may be associated with the response to ICI therapy.

Guo et al. identified exhausted T cell clusters by scRNA-seq, and these exhausted T cells were mostly populated together with cells from the tumors [31]. In line with this, a strong *drebrin1* expression was observed in cells from the tumors. Moreover, *drebrin1* expression was prominent in exhausted tumor-infiltrating CD8^+^ T cells. In contrast, exhausted tumor-infiltrating CD4^+^ T cells barely expressed *drebrin1*, suggesting that drebrin is an exhaustion-associated molecule distinct from other markers, such as PD-1, TIM-3, LAG-3, and CXCL13.

Our study has several limitations. The survival analysis was retrospective, with a small patient cohort, implying a limited number of events. Moreover, only patients with Sq were involved in this study. Further investigation based on a large cohort of patients, including lung adenocarcinoma patients, is therefore recommended. Additionally, the analysis of drebrin^+^ T cells using clinical samples, such as TILs, from resected tumors is necessary to reveal the role of the drebrin^+^ T cells. A limited number of immune subsets were analyzed in this study. Thus, other tumor-infiltrating immune subsets must be analyzed.

In conclusion, our study suggests that drebrin-expressing T cells present an exhausted phenotype and that drebrin-expressing TILs affect clinical outcomes in patients with resectable squamous cell lung cancer.

## 4. Materials and Methods

### 4.1. Patient Population and Tissue Sampling

Surgical tissue specimens were collected from 34 patients with Sq who underwent curative surgery between 1 January 2013 and 31 July 2014 at the Department of Thoracic and Breast Surgery of Kumamoto University Hospital. A total of 32 men and 2 women were included in this study. The median age was 70.5 (range 55–79) years old. The patients were enrolled after obtaining informed consent for the use of their biological samples for research purposes. The tissue sample collection was approved by the Kumamoto University Institutional Review Board (IRB number: 402; approval date: 11 November 2019). The tumor stage was determined using the 7th TNM staging system. The retrospective analysis of the drebrin^+^ TILs was also approved by the Kumamoto University Institutional Review Board (IRB number: 2349; approval date: 19 July 2021), which waived the need to obtain informed consent because the data were analyzed retrospectively and anonymously. This study was conducted in accordance with the principles of the Declaration of Helsinki.

### 4.2. Fluorescent Multiplex Immunohistochemistry

Fluorescent multiplex immunohistochemistry was performed with OPAL multiplex fluorescent immunohistochemistry reagents (PerkinElmer, Waltham, MA, USA), as previously described [6,7,25]. Formalin-fixed and paraffin-embedded tissue sections were treated to remove the paraffin, hydrated, and exposed to microwave radiation in 10 mM sodium citrate buffer (pH 6.0) for 10 min. The sections were incubated with 3% H_2_O_2_ for 5 min to inhibit the endogenous peroxidase activity, washed with 0.05% Tween 20 in Tris-buffered saline (TBST), exposed to blocking buffer (5% goat serum and 0.5% bovine serum albumin (BSA) in phosphate-buffered saline (PBS)) for 20 min, and incubated for 60 min with primary antibodies, including mouse anti-drebrin, produced as previously described [43], rabbit anti-CD3 (ab16669, clone SP7; Abcam, Cambridge, UK), and rabbit anti-pan-cytokeratin (ab86734, clone AE1/AE3 + 5D3; Abcam). They were then washed with TBST, incubated with horseradish peroxidase (HRP)-conjugated anti-mouse (Nichirei, Tokyo, Japan) or anti-rabbit (Nichirei) secondary antibodies for 10 min, and washed again, and the immune complexes were detected using OPAL reagent. The nuclei were counterstained with 4′,6-diamidino-2-phenylindole (DAPI) (Dojindo, Kumamoto, Japan) in PBS, and whole sections were mounted on ProLong Diamond (Thermo Fisher Scientific, Waltham, MA, USA). The multiplexed slides were observed with a fluorescence microscope (BZ-X700; Keyence, Osaka, Japan).

### 4.3. Quantification of TILs

The TILs were quantified as previously described [6,7]. Briefly, the high-speed scanning of all the cytokeratin-positive areas was performed on the whole slides with BZ-X 700 using a 20× objective lens. Images of the full cytokeratin-positive areas were analyzed using StrataQuest (TissueGnotics, Vienna, Austria). The size and staining intensity of the DAPI and pan-cytokeratin were adjusted for nuclear and tumor detection. The cutoff thresholds for CD3 and drebrin were determined by two independent observers. Finally, all the images were automatically analyzed by StrataQuest, generating statistics including the numbers of CD3^+^ cells and CD3^+^/drebrin^+^ cells. The localization of the TILs was classified as being within the tumor cell nest or the surrounding stroma according to their relationships with the tumor cells. Cells localized inside the area of the pan-cytokeratin-positive tumor cells were recognized as TILs within the tumor cell nest; otherwise, the cells were recognized as TILs within the surrounding stroma. At the time of the quantitative analysis, the clinical information was blinded.

### 4.4. T Cell Stimulation In Vitro

Blood samples from healthy donors were collected in cell preparation tubes containing sodium citrate (BD Vacutainer CPT tubes; BD Biosciences, Franklin Lakes, NJ, USA). Peripheral blood mononuclear cells were obtained by centrifugation following the manufacturer’s protocol. The CD3^+^ T cells were then purified by magnetic-activated cell sorting technology using anti-CD3 beads, according to the manufacturer’s instructions (Miltenyi Biotec, Bergisch Gladbach, Germany). The purity of the CD3^+^ T cells exceeded 98%. The purified T cells were cultured at 2 × 10^5^ cells on 24-well plates using RPMI 1640 medium (Wako, Osaka, Japan) supplemented with 10% fetal bovine serum (Biological Industries, Kibbutz Beit Haemek, Israel) at 37 °C in 5% CO_2_. The cells were stimulated with CD3/CD28/CD2 beads (T cell Activation/Expansion Kit; Miltenyi Biotec) for up to 96 h, and western blot, immunofluorescence, and flow cytometric analyses were performed. Written informed consent was obtained from all the healthy donors involved in this assay, and the in vitro analysis was approved by the Kumamoto University Institutional Review Board (IRB number: 2349; approval date: 19 July 2021).

### 4.5. Western Blot Analysis

Western blot analysis was performed on the T cells cultured in vitro, as previously described [44]. The cells were washed with PBS, lysed with sodium dodecyl sulphate (SDS) sample buffer (2% SDS, 10% glycerol, 50 mM Tris-HCl (pH 6.8)), mixed with bromophenol blue and dithiothreitol (final concentration, 100 mM), and incubated at 95 °C for 5 min. The lysates (5 µg of protein) were then fractionated by SDS-polyacrylamide gel electrophoresis on a 5–20% gradient gel (Pagel; Atto, Tokyo, Japan), and the separated proteins were transferred to a nitrocellulose membrane (Hybond; GE Healthcare, Boston, MA, USA). The membrane was incubated with primary antibodies, including mouse anti-drebrin and mouse anti-β-actin (A5316, clone AC-74; MilliporeSigma, Burlington, MA, USA). The immune complexes were detected using HRP-conjugated anti-mouse secondary antibodies (NA931VS; GE Healthcare, Boston, MA, USA) and enhanced chemiluminescence reagents (ImmunoStar LD; Wako, Osaka, Japan). Images were obtained using ChemiDoc Touch (Bio-Rad, Hercules, CA, USA).

### 4.6. Immunofluorescence Staining

Immunofluorescence staining was performed on the T cells cultured in vitro, as previously described [45]. The washed cells were fixed with 4% formaldehyde for 10 min at 4 °C and washed with PBS. The suspension of fixed cells was immobilized onto glass slides using a cytospin. The slides were then exposed to blocking and permeabilizing buffer (5% goat serum, 0.25% Triton X-100, and 0.5% BSA/PBS) and stained with primary antibodies including mouse anti-drebrin and rabbit anti-CD3 (ab16669, clone SP7; Abcam) for 60 min. They were then washed with 0.2% Triton X-100/PBS and incubated with secondary antibodies (anti-mouse Alexa Fluor 488 (A32732) and anti-rabbit Alexa Fluor 555 (A32723), both from Thermo Fisher Scientific). The nuclei were counterstained with DAPI (Dojindo) in PBS, and the whole sections were mounted on ProLong Diamond (Thermo Fisher Scientific). The slides were observed using a confocal fluorescence microscope (FV3000; Olympus, Tokyo, Japan).

### 4.7. Flow Cytometric Analysis

Multi-parameter flow cytometric analysis was performed on the T cells cultured in vitro, as previously described [46,47,48]. The washed cells were incubated with an Fc-receptor-blocking agent (Militenyi Biotec) and stained with surface antibodies for 20 min at 4 °C in the dark. Intracellular staining was performed using a Foxp3 fixation kit (eBioscience, San Diego, CA, USA) following the manufacturer’s protocol. The cells were stained with intracellular antibodies for 40 min at 4 °C in the dark. The following monoclonal antibodies were used: APC-CD3 (clone UCHT1), APC/Fire750-CD4 (clone A161A1), PerCP/Cy5.5-CD8a (clone RPA-T8), PE/Cy7-PD-1 (clone EH12.2H7), Brilliant Violet 421-TIM-3 (clone F38-2E2), Alexa Fluor 488-LAG-3 (clone 11C3C65) (all from BioLegend, San Diego, CA, USA) and APC-CXCL13 (clone 53610; Thermo Fisher Scientific). Anti-drebrin antibody was conjugated with R-PE using an Ab-10 Rapid R-Phycoerythrin Labeling Kit (Dojindo). Matched isotype controls were used for each antibody to establish the gates. The live cells were discriminated using LIVE/DEAD Fixable Aqua Dead Cell Stain (Thermo Fisher Scientific), and dead cells were excluded from all the analyses. All the flow cytometric analyses were performed using a BD FACSVerse (BD Biosciences, Franklin Lakes, NJ, USA). The data were analyzed using FlowJo software version 10.8.1 (FlowJo LLC, Ashland, OR, USA).

### 4.8. Gene Expression Analysis Using Published Single-Cell RNA Sequence (scRNA-Seq) Database

The single-cell data for *drebrin1*, *PDCD1*, *HAVCR2*, *LAG3*, and *CXCL13* were downloaded from http://lung.cancerpku.cn (accessed on 4 April 2022) [31] and analyzed using R version 4.2.1 (the R Foundation for Statistical Computing, Vienna, Austria). Additionally, we analyzed the expression of these genes at http://scdissector.org/leader (accessed on 11 May 2022) [32].

### 4.9. Statistical Analysis

The patient characteristics were described according to the status of drebrin^+^ T cell infiltration. Fisher’s exact test for the categorical data and the Wilcoxon rank-sum test for the continuous data were used. We presented the patient characteristics as medians. The relapse-free survival (RFS) and overall survival (OS) were evaluated using the Kaplan–Meier method, and the differences were estimated using two-tailed log-rank tests. The RFS was measured from the date of surgery to that of documented progression or death owing to any cause. The OS was measured from the date of surgery to that of death. Recurrence was determined by imaging and/or clinical observation according to judgment of the treating oncologist. Patients who did not progress or die by the last follow-up were censored. The data cutoff date was three years after surgery. The survival analysis was conducted using univariate analyses and multivariate Cox proportional hazard regression models, using propensity scores to correct potential confounding factors that may affect the treatment assignment. For the multivariable modeling, we used propensity score adjustments for age, sex, smoking history, pathological stage (I–II or III), adjuvant therapy, pleural invasion, lymphatic invasion, vascular invasion, and the rate of drebrin^+^ tumor cells. Each factor was categorized as shown in Table 1. The propensity score adjustment method preserves the statistical power by reducing the covariates to a single variable. To evaluate the adjusted effect of drebrin^+^ T cell infiltration, propensity scores were estimated through a binary logistic regression, providing the predicted probability with a high or low infiltration of drebrin^+^ TILs with a function above the background factors. Next, we performed survival analyses using multivariate Cox proportional hazard models with inverse probability of treatment weighting (IPTW), using the propensity score to balance the relevant characteristics between the high or low drebrin^+^ T cell infiltration groups. To confirm the statistical robustness, we utilized another method, using the propensity score as a covariate in multivariate Cox proportional hazard models. The in vitro data are presented as box-and-whisker plots. The lines indicate the median values. The box interquartile range (IQR) values and whiskers were 1.5 × IQR values, as calculated by Tukey’s test. The means of two groups were compared using the Wilcoxon rank-sum test, and the means of three groups were compared by one-way analysis of variance with Bartlett’s and Tukey’s tests. Two analytical steps were performed to investigate the relationship between the drebrin expression in the T cells and triple-positive T cells. In the first step, the time trend of the proportion of drebrin^+^ or triple-positive T cells was arranged as a linear mixed-effect model with a random intercept and slope. Next, the slope of each individual was estimated in this model, and we characterized the value as the time trend of the proportion of each subset. In the second step, Spearman’s correlation coefficient was used to examine the relationship between the subsets. The data from the in silico analysis are presented as violin plots. The means of two groups were compared using the Wilcoxon rank-sum test, and the means of three groups were compared using Kruskal–Wallis and Dunn’s multiple comparison tests. The statistical analyses were performed using R version 4.1.3 or GraphPad Prism version 9.1.2 (GraphPad Software, San Diego, CA, USA). Statistical significance was set at *p* < 0.05.

## Figures and Tables

**Table 1:** Baseline characteristics of 34 patients with Sq undergoing curative surgery stratified by the infiltration of drebrin^+^ TILs.

	Total	High Drebrin^+^ T Cell Infiltration	Low Drebrin^+^ T Cell Infiltration	*p* Value
	N = 34	N = 17	N = 17	
Age, median (range)	70.5 (55–79)	71 (56–79)	70 (55–78)	0.71
Sex, N (%)				
Male	32 (94%)	17 (100%)	15 (88%)	0.48
Female	2 (6%)	0 (0%)	2 (12%)	
Smoking history, N (%)				
Current/former	33 (97%)	17 (100%)	16 (94%)	>0.99
Never	1 (3%)	0 (0%)	1 (6%)	
Brinkman index, median (range)	1385 (0–2760)	1500 (600–2000)	920 (0–2760)	0.38
Pathological stage, N (%)				
I–II	27 (80%)	13 (77%)	14 (82%)	>0.99
III	7 (20%)	4 (23%)	3 (18%)	
EGFR mutation status, N (%)				
Wild-type	32 (94%)	16 (94%)	16 (94%)	0.37
Mutant	1 (3%)	1 (6%)	0 (0%)	
Unknown	1 (3%)	0 (0%)	1 (6%)	
Adjuvant therapy, N (%)				
+	10 (29%)	4 (24%)	6 (35%)	0.71
−	24 (71%)	13 (76%)	11 (65%)	
Pleural invasion, N (%)				
+	6 (18%)	2 (12%)	4 (24%)	0.66
−	28 (82%)	15 (88%)	13 (76%)	
Lymphatic invasion, N (%)				
+	10 (29%)	6 (35%)	4 (24%)	0.71
−	24 (71%)	11 (65%)	13 (76%)	
Vascular invasion, N (%)				
+	13 (38%)	7 (41%)	6 (35%)	>0.99
−	21 (62%)	10 (59%)	11 (65%)	

Tumor stage was determined using the 7th TNM staging system. The regimens used for adjuvant therapy included cisplatin + vinorelbine (N = 4), cisplatin + gemcitabine (N = 1), carboplatin + paclitaxel (N = 2), and tegaful/uracil (N = 3). EGFR, epidermal growth factor receptor; Sq, squamous cell cancer; TILs, tumor-infiltrating T cells; TNM, tumor, node, and metastasis.

## Data Availability

The data generated in this study are available upon request from the corresponding author. The gene expression profile data analyzed in this study were obtained from: http://lung.cancerpku.cn (accessed on 4 April 2022) and http://scdissector.org/leader (accessed on 11 May 2022).

## Supplementary Materials

The following supporting information can be downloaded at: https://www.mdpi.com/article/10.3390/ijms232213723/s1.

## Abbreviations

CI, confidence interval; CXCL13, chemokine ligand 13; EGFR, epidermal growth factor receptor; HR, hazard ratio; ICI, immune checkpoint inhibitor; IQR, interquartile range; ITPW, inverse probability of treatment weighting; LAG-3, lymphocyte activation gene 3; MAIT, mucosal-associated invariant T cells; mIF, multiplexed immunofluorescence; mIHC, multiplex immunohistochemistry; NSCLC, non-small-cell lung cancer; OS, overall survival; PD-1, programmed cell death-1; RFS, relapse-free survival; scRNA-seq, single-cell RNA sequence; Sq, squamous cell cancer; TILs, tumor-infiltrating lymphocytes; TIME, tumor immune microenvironment; TIM-3, T cell immunoglobulin- and mucin-domain-containing protein 3; TNM, tumor, node, and metastasis; t-SNE, t-distributed stochastic neighbor embedding.
