# Supplementary material for: Clinical Implications and Molecular Characterization of Drebrin-Positive, Tumor-Infiltrating Exhausted T Cells in Lung Cancer
Source: Int J Mol Sci. 2022 Nov 8;23(22):13723. doi: 10.3390/ijms232213723 (PMC9694580; doi:10.3390/ijms232213723)
Supplement: Supplementary file 1 [file ijms-23-13723-s001.zip › ijms-2008696-supplementary.pdf]

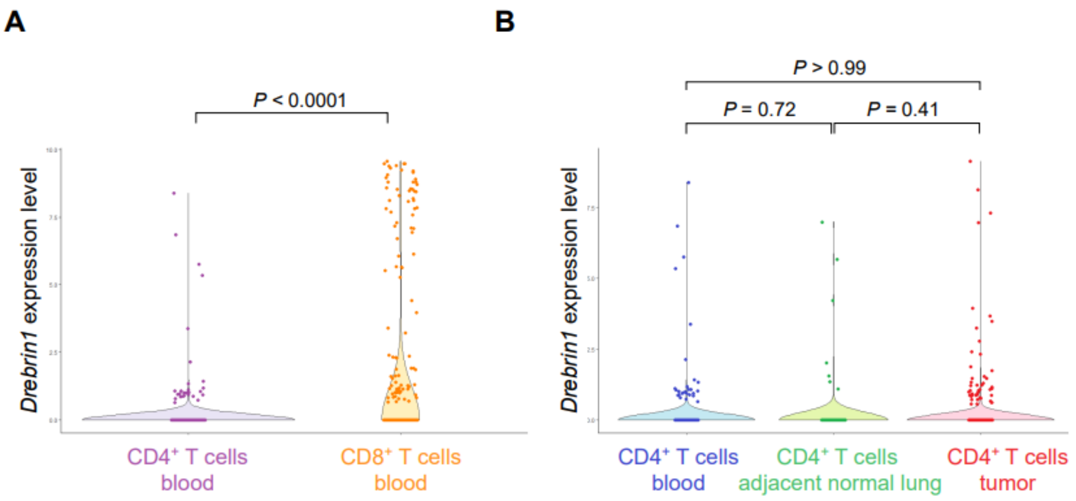

Figure S1. Difference in *drebrin1* expression among NSCLC patients. (**A**) Comparison of *drebrin1* expression between $CD4^+$ and $CD8^+$ T cells from tumor. Each dot represents one cell. (**B**) Comparison of *drebrin1* expression among $CD4^+$ T cells of different tissue samples. Each dot represents one cell. NSCLC, non-small cell lung cancer.

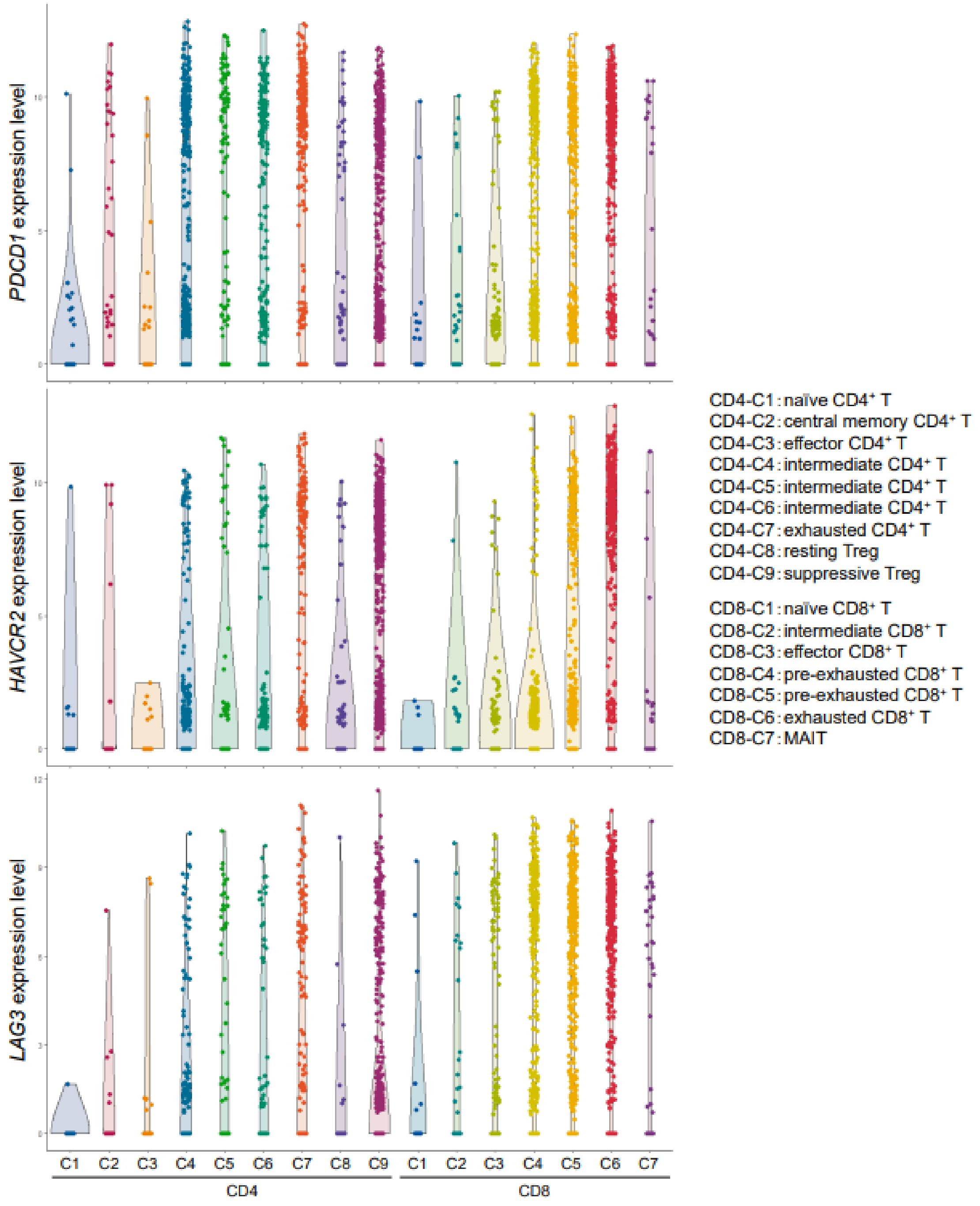

Figure S2. Expression of exhaustion-associated molecules. Expression of *PDCD1*, *HAVCR2*, and *LAG3* in each cluster was illustrated in violin plots. Each dot represents one cell. Definition of each cluster is indicated at the right panel; intermediate cells represent cells bridging naïve, effector, and exhausted clusters. MAIT, mucosal associated invariant T cells.
